# Assisted Reproductive Technologies: A New Player in the Foetal Programming of Childhood and Adult Diseases?

**DOI:** 10.3390/pediatric16020029

**Published:** 2024-04-26

**Authors:** Gavino Faa, Mirko Manchia, Vassilios Fanos

**Affiliations:** 1 Department of Medical Sciences and Public Health, University of Cagliari, Monserrato, 09042 Cagliari, Italy; gavinofaa@gmail.com; 2 Department of Biology, College of Science and Technology, Temple University, Philadelphia, PA 19122, USA; 3 Department of Pharmacology, Dalhousie University, Halifax, NS B3H 4R2, Canada; 4 Unit of Psychiatry, Department of Medical Sciences and Public Health, University of Cagliari, 09124 Cagliari, Italy; 5 Neonatal Intensive Care Unit, AOU Cagliari and Department of Surgical Sciences, University of Cagliari, Monserrato, 09042 Cagliari, Italy; vafanos@tiscali.it

**Keywords:** ART, ICSI, IVF, embryo transfer, foetal programming

## Abstract

Assisted reproductive technology (ART) is an emerging field in medicine that incorporates complex procedures and has profound ethical, moral, social, religious, and economic implications not just for the individuals who have access to this method but also for society. In this narrative review, we summarise multiple aspects of ART procedures and the possible consequences on the mother and newborn. Moreover, we provide an overview of the possible long-term consequences of ART procedures on the health of newborns, although longitudinal evidence is particularly scant. Users should be informed that ART procedures are not risk-free to prepare them for the possible negative outcomes that may occur in the perinatal period or even in childhood and adulthood. Indeed, risk estimates point to increased liability for major nonchromosomal birth defects; cardiovascular, musculoskeletal, and urogenital (in male newborns) defects; and any other birth defects. Less certainty is present for the risk of neuropsychiatric sequelae in children conceived through ART. Thus, its application should be accompanied by adequate counselling and psychological support, possibly integrated into specific multidisciplinary clinical programmes.

## 1. Introduction

Assisted reproductive technology (ART) is an emerging field in medicine that incorporates complex procedures and has profound ethical, moral, social, religious, and economic implications not just for the individuals who have access to this method but also for society. There are several important open questions regarding ART that have been discussed in recent years in the literature. For instance, it remains unclear who maintains the property of the embryos. Also, there is also no specific indication of how many embryos should be developed for each woman undergoing ART. Furthermore, the upper age limit for women asking to access ART procedures is still a matter of debate. Finally, there is still uncertainty on whether same-sex couples and single women could be eligible for ART. These aspects emphasise the numerous implications emerging from the field of assisted fertilisation in infertile or sub-fertile couples, with opinions changing and evolving in parallel with society. Further, the psychological and emotional burden associated with ART is substantial. Women asking for ART might show complex emotional feelings and be ready to face all the risks underlying the procedures of ART since they are extremely focused on the outcome, i.e., the ability to deliver their own daughter/son, which will change their lives. However, psychological well-being is essential during ART procedures. For instance, a recent study showed that women with recurrent implantation failure showed high serum epinephrine levels, which might result from psychological distress and high anxiety levels [1]. Importantly, epinephrine signalling was shown to regulate endometrial receptivity through the PI3K-AKT and FOXO1 pathways [1].

In this context, clinicians asked about the application of fertility technologies that provide couples with all the information regarding the complexity of the procedures as well as the possible risks during pregnancy for the woman and newborn(s) in the perinatal period. Moreover, women asking for ART should be informed in detail about possible risks regarding a higher susceptibility of the offspring to develop diseases in childhood and later in life. Thus, the overarching aim of this narrative review was to better delineate the long-term risks associated with ART for the offspring of mothers undergoing these procedures. To this end, we summarise multiple aspects regarding ART procedures and the possible consequences for the mother and newborn. Moreover, we provide an overview of the possible long-term consequences of ART procedures on the health of newborns, although longitudinal evidence is particularly scant. In the final part of this work, we discuss the role of ART in the foetal programming of adult diseases, according to Barker’s theory [2].

## 2. Historical Notes

The first baby conceived with ART was born through caesarean section following a full-term pregnancy on 25 July 1978. Louise Brown, then defined as a “baby miracle” or “superbabe” on the first page of the Evening News is now a 45-year-old woman and a mother of children. In 2010, the English scientist Robert Edwards was awarded the Nobel Prize in Physiology and Medicine for the development of human in vitro fertilisation (IVF). Edwards’ activity started in the 1960s of the last century, working on the removal of an egg from a woman and the maturation in vitro of oocytes [3]. Further studies focused on fertilisation in a test tube of oocytes matured in vitro [4] and the in vitro fertilisation of human oocytes matured in vivo [5]. The activity of Edwards and his co-workers eventually ended with placing a fertilised egg into a woman until the birth of a newborn after reimplantation of the embryo [6]. The long research project of Robert Edwards and Patrick Steptoe, defined “a bumpy road to human in vitro fertilization” [7], has made possible the treatment of infertility, a condition afflicting a large number of couples, ranging from 5% up to more than 10% in different countries worldwide. A new field of medicine has emerged thanks to the work of Robert Edwards, and his contributions represent a milestone in modern medicine [8]. Epidemiological data collected by the Centers for Disease Control (CDC) show that in 2010, 147,260 ART procedures were performed in the USA, with 47,090 live birth deliveries and 61,564 ART-related newborns. Worldwide, more than 10 million children have been born with ART, comprising about 8% of children born in Europe and about 5% of children born in the USA in 2018 [9].

## 3. Concepts of ART

### 3.1. Different Techniques Used for ART

Assisted reproductive technology (ART) includes multiple fertility treatments in which eggs or embryos are handled to promote successful pregnancies and healthy newborns. Assisted reproduction is based on transfer into the womb of fresh or frozen embryos, with significant differences due to the cryopreservation on the embryo [10]. Moreover, the embryo culture medium may influence the perinatal outcome [11].

### 3.2. Health and Development in Children Conceived through ART

Women undergoing ART procedures are more likely to deliver multiple-birth infants because more than one embryo is transferred during the procedure. Multiple births may pose substantial risks to mothers and newborns, including an increased incidence of low-birth-weight infants in ART-conceived pregnancies [12]. Follow-up of children conceived through ARTs revealed an increased incidence of multiple complications, including foetal hypotrophy, preterm delivery, and neonatal complications. Moreover, a higher incidence of congenital malformations was reported in ART-conceived babies [13].

A study on maternal and perinatal outcomes carried out in spontaneous versus assisted-conception twin pregnancies in Italy between January and June 2011 confirmed the presence of ART-related effects in neonates [14]. Specifically, the authors found that gestational age and birth weight were lower in the ART group, but, conversely, preterm delivery, placental abruption, and gestational diabetes were higher in the ART group [14]. In addition, respiratory complications in the perinatal period, patent ductus arteriosus frequency, and admission to NICU centres were higher among ART-conceived newborns compared to spontaneously conceived neonates [14]. The Italian study confirmed that twin pregnancies conceived via ART were at a greater risk of poorer maternal and neonatal outcomes than spontaneous twin pregnancies, suggesting that these effects might be related to the type of conception and/or specific negative features of subfertile parents undergoing infertility treatments.

A subsequent study carried out on 6420 dichorionic twins conceived through ART confirmed the higher frequency of preterm birth (75%), very preterm birth, low birth weight, placenta previa, elective caesarean section, and congenital malformations in twins conceived after ART [15]. In addition, a quantitative meta-analysis of 21 cohort studies showed that multiple pregnancies achieved with IVF/ICSI carried a significantly higher risk of chromosomal defects and urogenital and circulatory system malformations, but not of other typical congenital malformations such as cleft lip and/or palate, eye, ear, face, and neck and respiratory, musculoskeletal, nervous, or digestive system malformation [16]. Interestingly, children conceived through ART grew differently than spontaneously conceived children in early life, reaching the same weight and height by the age of 17 years [17]. Specifically, ART children were born smaller but grew faster after birth, resulting in increased height and weight at the age of 3 years, while, in subsequent years, the difference in height and weight from naturally conceived children diminished until, at age 17 years, no differences were evident [17,18]. Importantly, elements of the ART procedure contributed to the differences in growth patterns as children conceived by frozen embryo transfer tended to be larger at week 18 of pregnancy and continued to be bigger up to age six, but, again, no differences were present at age 17 years [17,18].

Among infants conceived with ART, 36.6% are born preterm, compared with 12% among all infants born in the general population. Moreover, 6.6% of all ART newborns are very preterm, compared with 2% among neonates in the general population. In Australian women, ART-conceived newborns were less likely to be born at term and had lower birth weights [19]. In the same study, no difference was found between ART and non-ART newborns regarding the incidence of respiratory distress in the perinatal period and the incidence of admission to neonatal intensive care unit (NICU) centres. Interestingly, the odds of neonatal acidosis were significantly lower in the ART cohort compared with newborns conceived spontaneously [19].

Iatrogenic twinning is generally considered the main ART-related side effect. ART-related twin pregnancies are associated with an increased risk of preterm delivery, low and very low birth weight, and increased incidence of placenta praevia. In addition, the pregnancy may be complicated by a vanishing twin [20]. The number of embryos transferred in the same procedure was strictly related to the mother’s age: 2 in women aged <35 years; 2.4 in women aged 35–40 years; 3 among women aged more than 40 years. Among infants conceived with ART, 46% were born in multiple deliveries. Therefore, 20% of all multiple-birth infants and 19% of all infants were conceived with ART. Concerning triplet infants, the percentage of ART-related births was 33% [20].

Concerning singleton pregnancies, several studies show that ART procedures might increase the likelihood of adverse health outcomes. For instance, a recent meta-analysis [21] shows that singleton ART pregnancies have a higher risk of malpresentation compared to those conceived naturally. In addition, a study examining a large cohort from the Nordic registry showed that the risk of major malformations in live-born singletons was slightly higher after fresh ICSI compared with fresh IVF [22]. Finally, singletons appear to have a slighter higher risk of placenta previa compared to twin pregnancies [23], with estimates in both cases higher than those of naturally conceived pregnancies.

## 4. Clinical Outcomes of ART

### 4.1. ART and Adverse Pregnancy Outcomes

A study carried out on Australian women who conceived using ART evidenced that they were less likely to have a spontaneous vaginal delivery compared to control women of the general population. Moreover, the use of ART was associated with an increased rate of elective caesarean section, emergency caesarean section, and instrumental delivery [19]. A French narrative review summarising evidence published after 2010 showed that the preeclampsia rate was increased after frozen embryo transfer, especially in cases of artificial cycles, multiple pregnancies, and gamete donation [24]. Concerning the occurrence of placenta previa, data show that compared to spontaneous pregnancies, ART gestation is characterised by a larger placenta, with a higher placental weight/newborn birthweight ratio [25]. The increase in placental weight is independent of the ART method utilised and the length of gestation. Interestingly, a meta-analysis showed that ART procedures are a risk factor for placenta previa [23].

### 4.2. ART and Low Birth Weight

Among ART-conceived newborns, 31.6% are low birth weight, compared with 8% among all newborns. Infants conceived through ART comprise 5.6% of all low birth weight babies (<2500 g) and 5.6% of all very low birth weight (<1500 g) neonates. ART-conceived neonates are disadvantaged at birth compared with spontaneously conceived newborns, being characterised by lower birth weight, higher incidence of very low birth weight, and very preterm birth [26].

### 4.3. ART and Neonatal Death

The risk of neonatal and perinatal death is increased in ART neonates, clearly suggesting that ART-conceived babies are disadvantaged at birth compared with spontaneous pregnancies [26].

### 4.4. Congenital Malformations

Birth defects are significantly increased in newborns conceived with techniques based on ART. These procedures are indeed associated with a 30% increase in birth defects, compared to sub-fertile couples achieving pregnancy without ART characterised by a 20% increase in birth defects [27]. According to a study, the increase in birth defects observed in ART couples might be mainly due to the biological perturbations associated with infertility and less, if at all, to ART procedures. Based on the few studies on ART and congenital malformations, the risk of birth defects is not directly related to the ART procedure itself, depending on multiple associated factors. These include causes of infertility, maternal and paternal comorbidities, the age of the parents, the number of embryos, the state of maternal stress during gestation, and other probably unknown factors that interfere with the intrauterine environment [27]. A large population-based cohort study examined 135,051 ART children (78,362 singletons and 56,689 twins), 23,647 naturally conceived ART siblings (22,301 singletons and 1346 twins), 9396 children born to women treated with ovulation induction (6597 singletons and 2799 twins), and 1,067,922 naturally conceived children, comparing rates of birth defects [28]. The results showed that, compared to naturally conceived children, ART singletons (conceived from autologous oocytes, fresh embryos without the use of ICSI) had increased risks of major nonchromosomal birth defects, cardiovascular defects, and any other birth defect [28]. Further, compared to naturally conceived children, for ART singletons conceived (from autologous oocytes, fresh embryos) with the use of ICSI, risks were increased for major nonchromosomal birth defects, blastogenesis defects, and cardiovascular defects. In addition, the risk of musculoskeletal defects was increased as well as the risk of genitourinary defects in male infants [28]. In addition, another recent study aimed at defining a hierarchy of risks for pregnancy outcomes in women undergoing ART conception showed that major birth defects including cardiac, urogenital, and musculoskeletal defects are doubled after fresh intracytoplasmic sperm injection, the technique that accounts for about 70% of all fertilisation techniques [26].

### 4.5. Cardiovascular (Patent Ductus Arteriosus), Urogenital, and Musculoskeletal Defects

The association between ART and congenital heart defects (CHDs) was analysed in a study on 399 Japanese women undergoing assisted conception procedures [29]. In this study, women were subdivided into four groups according to the different procedures utilised: (1) 142 treated with ovulation-inducing agents; (2) 56 women submitted to artificial insemination by the husband; (3) 159 with IVF; (4) 42 women submitted to ICSI. In total, 17 out of 402 newborns (4.1%) showed severe CHDs requiring surgical treatment in the perinatal period or leading to death before one year of age. In the control group of women undergoing spontaneous conception, the incidence of congenital cardiac defects was 4.0%, suggesting that ART treatment is not associated with an increased risk of CHDs [29]. Male genital anomalies might be related to ART, especially hypospadias and cryptorchidism. Hypospadias is a condition characterised by the incomplete or ineffective formation of the urethral folds and affects about 0.3% of total live births [30]. Progesterone intake during pregnancy has been associated with an increased risk of developing hypospadias [31]. Silver et al. reported a five-fold increased risk for hypospadias in pregnancies after ART, probably due to increased exposure to progesterone [32]. In addition, Funke et al. reported a three-fold increased risk of hypospadias but not cryptorchidism [33]. Again, Bang et al. reported increased risks in the ART group for both hypospadias and cryptorchidism [34]. Interestingly, the authors associated the presence of urological defects with increased risks of pre-term birth and low birth weight. On the other hand, Aliani et al. reported no significant difference for any genital defect, especially among ART and ICSI [35]. Finally, a recent meta-analysis did not report an increase in the risk of musculoskeletal defects in children conceived with ART [36].

### 4.6. Neuropsychiatric Sequalae of ART

There is an ongoing debate on whether ART might influence neurodevelopment and modulate the lifetime risk of severe neuropsychiatric disorders. Several studies have attempted to explore this association using longitudinal cohort designs [37,38,39] and quantitative meta-analytical approaches [40,41,42]. Conflicting results have been reported regarding the childhood outcomes and, particularly, the cognitive outcomes in ART-conceived babies. Some authors reported an association with intellectual disabilities, including intellectual disability and an increased incidence of autism spectrum disorder (ASD) [39]. The study of Velez et al. [37] included a cohort of 1.3 million children and reported incidence rates (per 1000 person-years) of ASD equal to 1.9 among children in the unassisted conception group, 2.5 in the subfertility group, and 2.7 after fertility treatment. In general, the risk of ASD was slightly higher in children born to individuals with infertility, although this was mediated by obstetrical and neonatal factors [37]. Interestingly, the mother’s mean age was different in the different groups, ranging from 30.1 years in the unassisted conception group to 33.3 in the subfertility group and 35.8 years in the group of children conceived through ICSI of IVF. The last IVF or ICSI group was also characterised by a high incidence of multiple events, which might be correlated with the subsequent higher incidence of ASD: 29% of caesarean births, 78% of multifetal pregnancies, 50% of preterm births, and 25% of severe neonatal and perinatal morbidities [37]. In addition, Lo et al. [38] found that ICSI was associated with an increased risk of ASD and developmental delay in offspring whose parents experienced infertility in a cohort study of 1,575,971 singleton births. The risk of ASD also appeared to be increased in children born to women undergoing progesterone treatment during gestation [43]. Conversely, a study by Sandin et al. observed that the neurodevelopment of preterm infants with very low birth weight conceived through ART procedures did not differ from that of spontaneously conceived infants [44]. Similarly, in another study carried out on a large cohort of children conceived through ART, no increase in the incidence of disorders of the autism spectrum was observed in children born through ART compared with children born spontaneously to fertile women [45]. Regarding the different techniques utilised in ART, the risk of developing ASD later in life was greater in children conceived through ICSI compared with children conceived through IVF [46]. Overall, these data suggest that ART procedures might influence the physiologic neurodevelopment of embryos and foetuses through some epigenetic perturbations that might be explained in the context of the foetal programming modelling of adult neuropsychiatric disorders [47,48]. It should be noted that a meta-analysis did not show an increased risk of ASD in children born with ART procedures compared to those born to fertile women [40]. Finally, some studies focused on cerebral palsy. A meta-analysis found an increased risk of its development in children born through different ART treatments [42]. The highest risk, more than two-fold, was found to be associated with an increased risk of preterm birth and multiple births [49]. The increase in single embryo transfer (SET) over the period 1990–2014 followed by the decrease in multiple births was paralleled by a decrease in the incidence of cerebral palsy in ART-conceived children [50]. Finally, there is evidence that advanced paternal age at the moment of conception might not only increase the risk of genetic abnormalities, such as DNA mutations and chromosomal aneuploidies, and epigenetic modifications, such as the silencing of essential genes, but may also affect reproductive and fertility outcomes, such as the success rate of IVF and ICSI and premature birth rate [51].

## 5. ART and the Foetal Programming of Diseases in Childhood

Suggestive but still speculative data might explain the missing link between ART procedures and the increased risk of a plethora of diseases. Indeed, epigenetics is defined as the hereditary transmission of phenotypic changes due to modifications in gene expression that do not affect the DNA sequence but that are related to environmental changes. The most important molecular mechanisms underlying the epigenetic control of gene expression are DNA methylation at CpG nucleotides, covalent changes of histone proteins, and the remodelling of chromatin structures and non-coding RNAs. In recent years, a major role has been assigned to epigenetics in human development [52]. Physiological epigenetic changes are essential for cell function and differentiation during development, and some epigenetic modifications may be passed on from one parent to the offspring through gametes [53]. Consequently, epigenetic changes may have relevant consequences on human development and postnatal health [54]. Moreover, epigenetics plays a key role across the lifespan, influencing human health status across the entire course of human life [55].

Taken together, these data suggest that anything that interferes with physiological epigenetic reprogramming during embryogenesis can affect foetal gene expression, possibly determining important modulatory effects on the risks later in life regarding susceptibility to some diseases. These are the bases of the theory of foetal programming, the hypothesis proposed by Barker in the 1970s [2,56]. In this regard, it is plausible that all the procedures associated with ART, including ovarian stimulation, the exposure of embryos and gametes to an in vitro environment, and all the manipulations of embryos, may cause epigenetic changes that could modify the developmental trajectory of ART newborns during the lifespan [57,58]. According to these data, users should be informed that ART might carry some inherent risks (to date of unknown magnitude) of possible epigenetic reprogramming of the embryo, with possible consequences later in life [59]. Regarding the risk of cancer insurgence in children conceived through ART, an increase in childhood leukaemia has been reported, restricted to techniques based on the transfer of frozen embryos [60]. Moreover, an increased risk of liver tumours has been reported in children conceived through IVF procedures [61]. No significant increased risk of other cancers was identified in ART-conceived children compared with naturally conceived children [62]. It is conceivable, although speculative, that possible ART-induced modulation of foetal programming could exert effects on long-term health outcomes. Indeed, given that the first ART procedure was performed in 1978, the oldest ART-conceived woman is now in her early 40s. Even if the long-term effects of ART procedures cannot be fully assessed, the Barker hypothesis of the foetal programming of adult diseases might indicate that individuals conceived through ART have a higher risk of developing diseases. Interestingly, an increase in the incidence of arterial hypertension has been reported in ART-conceived children in their adolescence [63]. Moreover, metabolic disorders, type 2 diabetes, and cardiovascular diseases have been indicated as possibly associated with ART technologies [64]. These data indicate that the long-term impact of ART and its role in the foetal programming of adult diseases will become clearer in the future when ART-conceived individuals enter their middle and old age [65].

## 6. Discussion

ART represents a milestone in modern medicine and has decreased the impact of infertility, allowing many couples to reach parenthood. However, clinicians and users should be aware of the possible long-term negative health outcomes related to ART procedures. Thus, counselling for couples evaluating ART should include risk disclosure, when data are available and replicated, related to specific health outcomes. Users should be informed that ART procedures are not risk-free to prepare them for the possible negative outcomes that may occur in the perinatal period or even in childhood and adulthood. This is, in our opinion, a crucial aspect. Given that the oldest woman conceived by ART is now in her early 40s, no data are available on the possible detrimental effects of ART on conceived children developing diseases later in life. According to the theory of foetal programming, relevant changes in the intrauterine development caused by the complex procedures associated with ART may significantly change the embryonic and foetal environment, with possible consequences on multiple organs’ structure and function, which would only manifest later in life. However, these effects, if present, will be revealed in the future by the prospective cohorts so far collected. Paediatricians should include data regarding ART in the clinical records of their patients to programme follow-ups of ART-conceived children with the aim of reducing the risk of the onset of adult diseases [66]. Indeed, risk estimates derived from longitudinal cohort studies of ART-conceived individuals showed increased liability for major nonchromosomal birth defects; cardiovascular, musculoskeletal, and urogenital (in male newborns) defects; and any other birth defects. Less certainty is present on the risk of neuropsychiatric sequelae in children conceived through ART. These estimates could guide counselling for couples accessing ART procedures accompanied by adequate levels of psychological support.

In addition, assisted reproductive technologies are raising many social, ethical, religious, and legal implications that differ significantly across various nations, societies, and cultures [67]. The reduction in multiple births, including the numerous ART-related twin births, has been managed worldwide by reducing the number of embryos transferred per ART procedure in women of all age groups, associated with the promotion of single embryo transfer (SET) procedures, when clinically appropriate. Moreover, although ART contributes significantly to the increasing rates of multiple births, ART does not entirely explain this rise.

A final comment concerns the psychological impact that ART procedures might have on the offspring and their relationship with the parents. A recent review showed that the psychological adjustment of adolescents conceived via ART is not undermined by the manner of their conception and that they enjoy positive relationships with their parents, with no difference from those enjoyed by spontaneously conceived adolescents [68]. However, it remains unknown whether the development of a reproductive identity, i.e., how a person elaborates the context in which his/her conception originated, in adolescence is likely to influence interest in searching for or contacting donors, surrogates, and/or donor siblings [68].

## 7. Conclusions

In conclusion, ART procedures have successfully treated infertility and represent one of the cost-relevant successes of modern medicine. Risk estimates point to increased liability for major nonchromosomal birth defects; cardiovascular, musculoskeletal, and urogenital (in male newborns) defects; and any other birth defects. Less certainty is present on the risk of neuropsychiatric sequelae in children conceived through ART. Thus, its application should be accompanied by adequate counselling and education of the couples accessing ART procedures, allowing them to reach the best decision for their life and the lives of their children.
